# Two Sides of Meaning: The Scalp-Recorded N400 Reflects Distinct Contributions from the Cerebral Hemispheres

**DOI:** 10.3389/fpsyg.2013.00181

**Published:** 2013-04-23

**Authors:** Edward W. Wlotko, Kara D. Federmeier

**Affiliations:** ^1^Beckman Institute, University of IllinoisUrbana, IL, USA; ^2^Department of Psychology, University of IllinoisUrbana, IL, USA; ^3^Program in Neuroscience, University of IllinoisUrbana, IL, USA

**Keywords:** event-related potentials, N400, language, hemispheric asymmetry, hemispheric cooperation, sentential constraint, predictability, hierarchical linear modeling

## Abstract

The N400, a component of the event-related potential (ERP) associated with the processing of meaning, is sensitive to a wide array of lexico-semantic, sentence-level, and discourse-level manipulations across modalities. In sentence contexts, N400 amplitude varies inversely and nearly linearly with the predictability of a word in its context. However, recent theories and empirical evidence from studies employing the visual half-field technique (to selectively bias processing to one cerebral hemisphere) suggest that the two hemispheres use sentence context information in different ways. Thus, each hemisphere may not respond to manipulations of contextual predictability in an equivalent manner. This possibility was investigated by recording ERPs while presenting [in the left and right visual fields (VFs)] sentence-final words that varied over the full range of sentence-level predictability. RVF/left hemisphere items were facilitated (as evidenced by reduced N400 amplitudes) over a broader range of predictability compared with LVF/right hemisphere items, although both strongly predictable and completely unexpected items evoked similar responses in each VF/hemisphere. Further, the pattern of N400 amplitudes over the full range of predictability significantly differed from a linear response function for both VFs/hemispheres. This suggests that the N400 response recorded with standard central field presentation comprises different contributions from both cerebral hemispheres, neither of which on its own is sensitive to contextual predictability in an evenly graded manner. These data challenge the notion of a singular or unitary mode of comprehension and instead support the view that the left and right hemispheres instantiate unique, complementary language comprehension architectures in parallel.

## Introduction

One of the most salient aspects of brain organization is its division into two largely symmetric – but functionally complementary – cerebral hemispheres. The neurobiology of language has always been intertwined with the concept of hemispheric specialization, since the 1860s when the neurologists Broca and Wernicke characterized the profound language deficits that can result from damage to the left (but typically not right) hemisphere frontal and temporal brain regions that now bear their names. The asymmetry in patterns of brain damage that result in aphasia led to the classic view of language as a left hemisphere (LH) phenomenon, with the right hemisphere (RH) thought to contribute little if at all to typical language use (Ojemann, 1991). Neurocognitive models of language from the Nineteenth century to today have thus incorporated LH functional specialization as a core component (e.g., Geschwind, 1970, cf. Poeppel and Hickok, 2004; Federmeier, 2007).

Because of the striking language disturbances associated with LH-damage-based aphasia, more than 100 years passed before neuropsychologists systematically described more subtle, yet consistent language impairments caused by RH damage. Although basic language comprehension and production processes generally remain intact, RH patients often demonstrate difficulties with pragmatic or para-linguistic aspects of language, such as understanding humor, inferences, and thematic elements of narratives (Brownell et al., 1983; Gardner et al., 1983; Brownell and Martino, 1998). These findings ignited interest in the role of the RH in normal language functioning.

More recently, investigations of asymmetries in language processing have demonstrated different patterns of sensitivity or activation in the two hemispheres for word-, sentence-, and discourse-level information (Beeman and Chiarello, 1998). Much of this research has employed the visual half-field presentation technique, which relies on the contralateral organization of the visual system to induce hemispheric processing biases (see Banich, 2002 for a comprehensive discussion of the logic underlying the technique). In brief, information presented in one half of the visual field (VF) will be initially processed unilaterally in the contralateral visual cortex. This initial asymmetry in information delivered to the hemispheres has been shown to induce processing biases that can reveal functional asymmetries (which can be detected far downstream from the initial stimulation, see, e.g., Evans and Federmeier, 2007). The half-field methodology was first demonstrated in commissurotomy patients, in whom the two hemispheres are disconnected from each other (see Gazzaniga, 1970). Thus, information transmission through the cerebral commissures was largely prevented. Now decades of studies with healthy individuals show that robust and replicable asymmetries can be observed with the half-field technique even when the corpus callosum is intact – i.e., even when hemispheric communication is more available – substantiating the effectiveness of the procedure (see, e.g., Hellige, 1993; Hugdahl and Davidson, 2003 for volumes describing and reviewing VF research in multiple domains of cognition such as attention, emotion, memory, etc.). In all cases, behavioral and neurophysiological dependent measures are compared as a function of VF of presentation. Differences in patterns for the two VFs are taken as an indication of asymmetric hemispheric processing biases.

Robust hemispheric asymmetries for specific language subprocesses have been uncovered using the half-field technique. Differences have been proposed in the organization or activation of semantic memory (Jung-Beeman, 2005), in the time course with which information can be activated or processed (Koivisto, 1997), in both auditory and visual low-level word recognition processes (Hellige et al., 1989; Rayman and Zaidel, 1991), and in sensitivity to sentence-level, discourse-level, and pragmatic contextual information (Faust, 1998; Long et al., 2005). The body of work on hemispheric processing differences from visual half-field studies is broadly consistent with both neuropsychological studies contrasting LH and RH patients groups as well as with neuroimaging studies in these same domains (see, e.g., Bookheimer, 2002; Scott and Wise, 2004; Boemio et al., 2005; Xu et al., 2005; Eviatar and Just, 2006). Thus, language subprocesses have been divided into those that are LH-based (or biased) or RH-based (or biased), but comparatively little attention has been focused on how the two hemispheres may work cooperatively to jointly effect normal language comprehension.

For example, several early behavioral studies suggested that RH processing of meaning relies primarily on word level information, whereas the LH advantages for language processing make use of sentence-level (syntactic, propositional) processes to influence meaning construction (reviewed in Faust, 1998). More recently, behavioral studies have shown that both hemispheres can take advantage of message-level context information to affect word processing (Chiarello et al., 2001; Faust et al., 2003), but that they may do so in different ways. Thus, it is important to uncover how the two hemispheres, working together, support everyday language comprehension. As described next, electrophysiological studies have played an important role in beginning to delineate the individual and joint contributions of the two hemispheres to normal comprehension.

In healthy adults, electrophysiology has been central to unraveling cognitive and neurobiological questions about language comprehension. The event-related potential (ERP) is a measure of neural activity in response to a specific event of interest (e.g., presentation of a critical word within a sentence context). An ERP waveform represents this electrical brain activity summated at the scalp, recorded over time. Each waveform is composed of components that are reflections of specific cognitive processes of interest (see Fabiani et al., 2007). Its millisecond-level temporal resolution means that the ERP can capture the rapid and temporally overlapping processes involved in language comprehension. For example, different ERP components index perceptual, semantic, structural/syntactic, and pragmatic aspects of language, and many other types of cognitive processing involved in comprehension (see, e.g., Kutas et al., 2007).

One functionally specific marker of the processing of meaningful information is the N400, a negative-going wave in the ERP peaking around 400 ms post-stimulus onset. The N400 is part of the normal brain response to all meaningful and potentially meaningful stimuli in all modalities, including written and spoken words (Kutas and Federmeier, 2011). As such, the N400 has been particularly important for investigations into the construction of meaning from language. Thirty years of research has demonstrated the profound impact on meaning processing of context information of many types: lexico-semantic, sentential, pragmatic, background world knowledge, and even social contexts can all influence semantic processing as indexed by the N400 (reviewed in van Berkum, 2008).

A simple way to examine the effect of sentential context on message-level processing is to vary the predictability (or *constraint*) of the context. Kutas and Hillyard (1984) first demonstrated the strong relationship between N400 amplitude and the predictability of words within sentence contexts during online language comprehension. As predictability increases, N400 amplitude decreases. At the grand average level, this inverse correlation has a magnitude of about 0.9 (see also Wlotko and Federmeier, 2012a). The reduction in N400 amplitude is interpreted as reflecting the increasing facilitation of semantic processing for the critical word as a consequence of the rich contextual information available in increasingly constraining sentences. In particular, the N400 amplitude reduction, or *N400 effect*, is thought of as a reflection of reduced demands on semantic processing, similar to a reduced response time in behavioral priming paradigms.

The advantages of electrophysiology for investigating language, including its superb temporal resolution, the multidimensional nature of the measurements, and the lack of a requirement to impose a secondary task other than reading for comprehension (see, e.g., Van Petten, 1995 for a detailed argument regarding the advantages of omitting an overt online task from investigations of comprehension), make it an illuminating tool for investigating hemispheric contributions to comprehension. Indeed, given that the two hemispheres may contribute differently to message-level comprehension, and that the N400 is likely generated in widespread brain areas across both hemispheres, determining how the two hemispheres contribute to this well-studied brain response would represent an important advance in the understanding of neural mechanisms of language.

A main disadvantage of ERP methodologies is the limited usefulness for identifying sources of brain activity recorded at the scalp. This can be true even for somewhat localized generators of neural activity, but is especially true for components like the N400 that are likely to be generated in a widely distributed network of brain regions. Particularly in such cases, even high-density ERP recording systems that offer a more detailed mapping of electrical fields at the scalp are not able to unambiguously determine a unique set of brain areas that generate scalp-recorded components of interest (Luck, 2005). Although converging evidence from intracranial recordings and other neuroimaging modalities suggests that the generators of the N400 (and N400-like effects) probably include anterior medial temporal lobe, superior temporal gyrus, and perhaps contributions from some parietal and frontal areas, there is currently no complete description of the neuroanatomical basis of the N400 (see Van Petten and Luka, 2006 for a review).

In light of this limitation, some prior studies have exploited the advantages of electrophysiological techniques in combination with visual half-field methods to investigate hemispheric asymmetries in brain responses during language comprehension. As for behavioral tasks, patterns of dependent measures (e.g., N400 amplitudes) are compared as a function of VF. These studies have revealed that both hemispheres use sentential context to process message-level meaning (see Federmeier et al., 2008 for review), consistent with conclusions drawn from recent behavioral studies (e.g., Gouldthorp and Coney, 2009). However, each hemisphere may use the contextual information in a different way.

In one study (Wlotko and Federmeier, 2007), ERPs were recorded while sentence-final critical words were presented in either the left or right visual field (LVF or RVF). The critical words varied in predictability: they were strongly expected, weakly expected, or unexpected (but plausible) in the sentence contexts, determined by norming responses in a separate cloze probability task (in which participants are asked to complete a sentence frame with the word that first comes to mind; the proportion of responses for a particular word is that word’s cloze probability in that sentence frame). When words were presented in either VF, N400 responses were smaller for strongly expected words compared to unexpected words; this is the well-replicated N400 expectancy effect (“*He bought her a pearl necklace for her*” … “*BIRTHDAY*.” vs. “*COLLECTION*.”). The size of the N400 expectancy effect was statistically indistinguishable across VF of presentation (as demonstrated in all VF-ERP studies of sentential context use). These findings show that both hemispheres can use sentential context to facilitate semantic processing of message-level meaning.

However, across VF of presentation, N400 responses to the weakly expected endings differed. For RVF/LH presentation, weakly expected items were facilitated to the same degree as strongly expected items, as these two conditions were statistically indistinguishable (“*He bought her a pearl necklace for her*” … “*BIRTHDAY*.” vs. “*He looked worried because he might have broken his*” … “*ARM*.”). By contrast, N400s to weakly expected items presented in the LVF/RH were statistically indistinguishable from those of the *unexpected* items (“*He looked worried because he might have broken his*” … “*ARM*.” vs. “*COLLECTION*.”). As such, the N400 facilitation for weakly expected items compared to unexpected items was significantly larger for RVF/LH items, and the N400 effect for strongly expected items compared to weakly expected items was significantly larger for LVF/RH items. Thus, the pattern of N400 response to variations in predictability of sentence context information differed across the two hemispheres. Additionally, both hemispheres showed a pattern that departed from the evenly graded relationship between predictability and N400 amplitude observed with typical (central VF) presentation.

These findings raise the intriguing possibility that rather than manifesting as a unitary phenomenon, the typically recorded N400 reflects distinct contributions from each hemisphere in parallel. Indeed, when RVF/LH and LVF/RH ERPs were averaged together, the classic graded N400 pattern emerged. Although the N400 is likely to be generated by widely distributed cortical areas, this brain response is typically thought of as a single functional unit that represents attempted access to semantic memory (Kutas and Federmeier, 2000; Federmeier and Laszlo, 2009). Our recent findings suggest that the distinct processing biases of the two hemispheres combine to form the graded pattern of N400 amplitudes observed with typical scalp-recorded potentials.

Thus, we claim that not only do the LH and RH both contribute to comprehension, sometimes each in their own way, but also that everyday language comprehension arises through joint contributions from each hemisphere. In this study, the sensitivity of the two hemispheres to sentential context is directly explored by parametrically varying the constraint of the contextual material across the full range of possible values (measured with cloze probability) and examining N400 responses as a function of VF of presentation. We suggest that the systematic inverse relationship between N400 amplitude and sentence-level predictability arises out of unique contributions from the two hemispheres, neither of which on its own is responsive to expectancy/predictability in an evenly graded manner. Thus, based on prior studies, we expect a monotonic decrease in N400 response with increasing contextual constraints for both VFs/hemispheres. However, the two VFs/hemispheres are expected to differ in the responses for weakly constraining contexts in particular, and both VFs/hemispheres should depart from an evenly graded (linear) pattern of N400 amplitudes as a function of cloze probability. Specifically, RVF/LH N400s are expected to evidence a larger reduction in N400 amplitude for weakly expected endings (relative to unexpected items), representing a greater degree of facilitation for semantic processing at weak levels of constraint. In contrast, LVF/RH N400s for weakly expected items are predicted to be more similar to unexpected items until constraint becomes much stronger. To investigate these hypotheses, we first replicate Wlotko and Federmeier (2007) with a traditional ERP approach. We then examine brain responses over the full range of cloze probability using statistical procedures (item-level analysis and hierarchical linear modeling, HLM) that have recently been shown to provide a fine grained characterization of the use of sentential context when applied to electrophysiological measures (Wlotko and Federmeier, 2012a).

Finally, we examine post-N400 effects to look for hemispheric differences in later stage processes. Recent studies of sentence comprehension have investigated different types of post-N400 effects that vary in scalp distribution and as a function of the relationship between a critical word and its prior context (reviewed in Van Petten and Luka, 2012). One such effect, termed a late frontal positivity, has been observed in response to unfulfilled predictions engendered by constraining sentential context (Federmeier et al., 2007). Interestingly, Wlotko and Federmeier (2007) demonstrated that the frontal positivity effect is not observed with lateralized presentation to either VF/hemisphere. We suggested that the processes leading to the generation of the frontal positivity may require hemispheric cooperation and thus are not engaged when one hemisphere dominates processing. As the critical words completing the constraining contexts in the present study never disconfirm predictions, we would not expect to observe this late frontal ERP positivity here – and, indeed, no frontal positivity effect was seen for central presentation of these stimuli (Wlotko and Federmeier, 2012b). However, with central presentation of these stimuli, we observed a left-lateralized, negative-going response to moderately strongly constrained sentence completions (75–90% cloze) compared to the other constraint conditions (Wlotko and Federmeier, 2012b). An item-level analysis revealed that the effect was largest for sentences that were likely to have a strong alternative completion based on the cloze norming data. For example, the sentence “*Each night the campers built a huge*… ” was completed with the word “*fire*” by most participants in the norming task. However, a sizable minority completed the sentence with the word “*tent*.” Thus, the negativity was linked to processes engaged when an alternative reading of the sentence context may have been considered by participants in the experiment, which would sometimes be incompatible with the “best” or highest cloze completion presented in the experiment. In these circumstances, readers may reconsider or revisit the contextual material to appreciate the multiple possible interpretations of the context. Here, we will determine whether this type of effect is observed with lateralized presentation and will examine the post-N400 time window to look for any later asymmetric effects in the ERPs.

## Materials and Methods

### Participants

Twenty-four native English speakers participated in the experiment. Twelve participants were female and 12 were male. Age ranged from 18–21 years, with a mean of 18.9 years. All participants were right-handed by self-report and as assessed with the Edinburgh Handedness Inventory (Oldfield, 1971). Mean handedness score was 0.7; four participants reported at least one left-handed immediate family member. All participants were screened for history of neurological or psychiatric diagnoses, neuro-active medication taken in the previous 6 months, and exposure to languages other than English before age 5. No participant who met any of the exclusionary criteria was admitted to the study. Data were collected from 6 additional participants but were not analyzed due to excessive eye movements or other artifactual contamination during electroencephalogram (EEG) recording. Participants received course credit for their time. All volunteers provided written informed consent before participating in the study and all protocols were reviewed and approved by the Institutional Review Board at the University of Illinois.

### Materials

The experimental stimuli consisted of 300 sentences for which the final words varied continuously through the full range of cloze probability (range of 0–100%). The stimulus set was identical to that used in Wlotko and Federmeier (2012a,b). Cloze probability was determined through norming studies described extensively in several previously published reports (e.g., Federmeier et al., 2007; Wlotko et al., 2012). Fifty sentences with unexpected but plausible sentence endings were also taken from these prior studies. The sentence frames completed with unexpected endings were always weakly constraining (i.e., the cloze value of the word with the highest cloze probability for that frame did not exceed 42%), but the sentence-final unexpected endings were never the word with the highest cloze probability for the sentence frame. The actual cloze value for the unexpected words was near zero and did not exceed 10%. For the remaining 250 sentences, the sentence-final critical word was always the best completion (the word with the highest cloze probability for that frame). As such, the constraint of the sentence was never violated with a less predictable word in these sentences.

Fifty items were selected from the 90–100% cloze range to create a condition of very strong constraint; the contrast with the Unexpected words produces the well-studied N400 expectancy effect. The remaining stimulus materials were divided into 50-item bins (25 per VF in each of Unexpected, 10–30, 30–50, 50–75, 75–90%); within the six bins, sentence-final critical words did not differ in lexical characteristics of Kucera–Francis word frequency (mean = 114, SD = 3.7), length (mean = 5.0, SD = 0.3), word class (80% nouns), or concreteness (mean = 520, SD = 16.0), imageability (mean = 543, SD = 11.9), or familiarity (mean = 575, SD = 4.1) ratings (all retrieved from the MRC Psycholinguistic Database; Wilson, 1988). Because the characteristics were relatively homogenous, other groupings of cloze probability did not cause major differences in lexical characteristics within or across VF. In addition, cloze probability is not correlated with any of the lexical characteristics (all *r* values < 0.08). Examples of the sentence materials are presented in Table 1.

**Table 1:** **Examples of stimuli and summary characteristics (mean, and standard deviation in parentheses) for the six 50-item cloze probability bins described in the Materials and Methods section**.

Cloze Bin	Sentence	Primary cloze	Alternate cloze	Sentence length	Kucera–Francis frequency	Number of letters
90–100	The little girl refused to go to sleep until he told her a **story**.	94.2% (2.7%)	2.6% (4.8%)	10.0 (3.4)	118.4 (111.8)	4.7 (1.2)
	Tricia had never seen a spider get tangled up in its own **web**.	
	They saw the lightening and heard the **thunder**.	
75–90	He was cold most of the night and finally got up to get another **blanket**.	83.2% (4.2%)	5.2% (3.7%)	10.2 (4.4)	110.8 (103.5)	4.7 (1.1)
	After two hours of hard work they decided to take a short **break**.	
	I just had a new sound system installed in my **car**.	
50–75	She pulled her head out from under the faucet and reached for a **towel**.	64.6% (8.2%)	9.7% (5.5%)	9.7 (4.3)	115.0 (131.2)	5.1 (1.3)
	Jim was saving boxes for a friend who was **moving**.	
	Ida wanted to sing folk songs at the picnic, so she brought her **guitar**.	
30–50	He was caught stealing a **car**.	38.8% (5.0%)	11.2% (4.8%)	9.6 (2.8)	110.0 (167.7)	4.8 (1.4)
	The technician never comes to work before **noon**.	
	She wished she had brought something to **eat**.	
10–30	It was time to hang the new **pictures**.	21.7% (5.5%)	8.3% (4.6%)	9.7 (3.1)	118.5 (180.0)	5.2 (1.7)
	They went to see the famous **actor**.	
	The police had never seen a man so **drunk**.	
Unexpected	The candidate had spent most of his funds on **drugs**.	1.4% (2.0%)	1.7% (2.8%)	10.1 (3.5)	113.1 (148.8)	5.4 (1.4)
	They waited a long time to see the **grades**.	
	Rushing out he forgot to take his **camera**.	

All sentences were pseudorandomly assigned to two lists such that half of the words appeared in the LVF and half appeared in the RVF. Thus, each critical word was seen by each participant in either the LVF or RVF but not both, and all critical words appeared in both the LVF and RVF across all participants. The conditions for assigning the sentences to the lists ensured that an equal number of words from each of the 50-trial cloze probability ranges appeared in each VF. Also, because most of the critical words were best completions generated by participants in a norming study, some critical words completed more than one sentence in the experiment. These were assigned to opposite VFs, or, in the few cases that a word was repeated more than once, the appearances were distributed equally across the VFs. The sentences were pseudorandomly reordered twice, and half of the participants were presented with one order and half with the other order. The conditions for order of presentation ensured that no more than three trials in a VF appeared sequentially and that repeated words were not presented in close proximity to other presentations of the word. VF of presentation and order of stimuli were counterbalanced across participants, and an equal number of males and females received each counterbalancing combination.

### Procedure

Participants were seated in a dimly lit room 100 cm in front of a 21″ CRT computer monitor. Each trial began with a warning sign (several pluses in the center of the screen) presented for 500 ms; the duration of the blank screen between the warning sign and the first word of the trial varied randomly from 500 to 1200 ms to avoid averaging in slow potentials associated with anticipation of sentence onset. Each word appeared in a Helvetica 22-point font with black text on a white background. All words subtended an approximate maximum of 0.4° of vertical visual angle and an approximate maximum of 2.3° of horizontal visual angle. Sentences were presented word-by-word in the center of the screen, except for each sentence-final word, which was presented with its inner edge 2° to either the left or right of fixation. All words were presented for 200 ms and each interstimulus interval was 300 ms. A 2.5 s pause followed each trial, which was then followed by a display of the prompt “Please go on when you are ready.” Participants initiated the next trial with a button press.

A central fixation point remained on the screen throughout the entire experiment below the point where the words were presented. Participants were asked to minimize blinks, eye movements, and muscle activity while reading, and to maintain central fixation while words were presented laterally. They were instructed to read the sentences for comprehension while keeping in mind that they would be asked questions about what they had read at the conclusion of the experiment. The recording session began with a short set of practice sentences to acclimate the participants to the task situation. The main experimental session was divided into 10 blocks of sentences, with participants taking a short rest between each block.

An EOG calibration procedure was completed after the main experimental session. A capital letter X was presented in pseudo-random order 15 times in each of the following locations: 1°, 2°, and 4° to the left and right of fixation; no more than three consecutive trials appeared in the same location. These data were used to choose electrooculogram (EOG) artifact rejection thresholds (described in the EEG processing section).

After the recording session, participants completed a recognition test. A list of 60 sentences, each missing its final word, was presented to the participants. Half of the sentences were read during the experiment and half were taken from various cloze normings and were not presented during the experiment. Participants were asked to mark each sentence read during the experiment, and of those, to fill in the missing word that was read during the experiment. Of the sentences seen during the experiment, 5 came from each 50-trial cloze bin described above. Of the five sentences from each bin, either two or three sentences came from one VF or the other, alternating for each bin (i.e., for one set of participants for a particular bin, two sentence endings would have been presented in the LVF and three in the RVF and vice-versa for the remaining participants). Within the five sentences, the average actual cloze value corresponded to the mean of each experiment-wide bin, and word frequency did not differ among the conditions within the recognition test.

### EEG recording and processing

EEG was recorded continuously from 26 geodesically arranged sites on the scalp using silver-silver chloride electrodes embedded in an Electro-cap. The sites are Midline Prefrontal (MiPf), Left and Right Medial Prefrontal (LMPf and RMPf), Left and Right Lateral Prefrontal (LLPf and RLPf), Left and Right Medial Frontal (LMFr and RMFr), Left and Right Mediolateral Frontal (LDFr and RDFr), Left and Right Lateral Frontal (LLFr and RLFr), Midline Central (MiCe), Left and Right Medial Central (LMCe and RMCe), Left and Right Mediolateral Central (LDCe and RDCe), Midline Parietal (MiPa), Left and Right Mediolateral Parietal (LDPa and RDPa), Left and Right Lateral Temporal (LLTe and RLTe), Midline Occipital (MiOc), Left and Right Medial Occipital (LMOc and RMOc), and Left and Right Lateral Occipital (LLOc and RLOc). The position of the cap was determined by placing the MiPf electrode at 10% of the nasion-inion distance from the nasion, the MiOc electrode at approximately 10% of the distance from the inion, and the MiCe electrode halfway between the mastoid processes. The electrodes were referenced online to the left mastoid and later referenced offline to the average of the left and right mastoids. Eye movements were monitored using a bipolar recording of EOG with electrodes placed on the outer canthus of each eye. Blinks were monitored with an electrode placed over the infraorbital ridge of the left eye, referenced to the left mastoid. Electrode impedances were kept below 4 kΩ and signals were amplified with Sensorium amplifiers set at a bandpass of 0.02–100 Hz. EEG was sampled at 250 Hz and saved on a hard drive.

EEG records were examined and marked for electromyographic (EMG), EOG, or other artifactual contamination. Using data from the EOG calibration procedure described above, rejection algorithm thresholds corresponding to eye movements of 1°, 2°, and 4° from fixation were determined for each participant. In the epoch consisting of the 100 ms before stimulus onset through 200 ms post-stimulus onset (corresponding to the duration of critical stimulus presentation plus a 100 ms baseline), individual trials containing eye movement activity that exceeded the average of the 1° and 2° thresholds were rejected to ensure that participants maintained fixation during presentation of lateralized stimuli for trials included in the ERP averages. After 200 ms, individual trials containing EOG activity exceeding the 4° threshold were rejected in order to prevent EOG activity from contaminating the ERP records. The effectiveness of this procedure was assessed by ensuring that for each condition of each participant, the average voltage recorded in the horizontal EOG channel (i.e., in the ERP for each condition) during time epochs of interest did not exceed the average voltage levels of the 1° and 2° eye movement calibration trials. Also, no effects of experimental conditions within VFs were observed in the EOG channels when subjected to statistical analysis.

Artifactual trials containing eye blinks were corrected (see Dale, 1994 for the procedure) and added back into the EEG record for 15 of the 24 participants. The remaining artifactual trials (12% for RVF, 13% for LVF, across participants) were excluded from further analysis. ERPs were computed from 100 ms before the onset of critical words to 920 ms after. The data were referenced to the algebraic mean of the left and right mastoids, and averages of artifact-free ERPs were calculated for critical words in each of the cloze probability partitions, after subtraction of the 100 ms pre-stimulus baseline. When analyzing ERPs with repeated-measures ANOVAs, Electrode Site is included as a factor but main effects of Electrode are not reported and interactions with Electrode are reported only when of theoretical interest. For tests with more than 1 degree of freedom in the numerator, Huynh–Feldt corrected *p*-values are reported along with epsilon values. EEG epochs were also analyzed with hierarchical linear modeling procedures (HLM), “nested” for each individual participant (see, e.g., Goldstein, 2011; see also Wlotko and Federmeier, 2012a for more information about HLM in the context of ERP analysis). In all cases, mean amplitude measurements were taken after a digital bandpass filter of 0.2–20 Hz was applied.

## Results

### Recognition test

Two participants were given the wrong version of the recognition test and their performance was not analyzed here. The other participants correctly recognized an average of 15 sentences from the experiment (50%) and false alarmed to an average of 2.8 sentences (mean *d*′ = 2.26). Thus, participants were attending to the experimental material. In addition, subjects correctly filled in the final word from the experiment in an average of 12.0 sentences, and incorrectly filled in an average of 2.3 words. Most subjects filled in a word for every sentence marked as “old” (they were encouraged to do so unless they felt it would be a complete guess), but about 1/3 of the subjects left some words blank. These data are similar to the *d*′ and hit rates reported in Wlotko and Federmeier (2012a) for young subjects reading the same sentences with critical words presented centrally: in that study, mean *d*′ was 2.14 and the mean hit rate for correctly completed sentence endings was 15.3 words.

To assess the effect of constraint on hit rates for each VF, sentences were collapsed into three cloze probability bins (High Cloze: 75–100%, Medium Cloze: 30–75%, and Low Cloze: <30%) and subjected to a 2 (VF) × 3 (Cloze) repeated-measures ANOVA. There was an effect of condition [*F*(2,42) = 4.98, *p* = 0.012, ε = 0.899], as participants correctly recognized high cloze probability sentences most often (3.0 out of 5 items for LVF, 3.4 for RVF), medium cloze sentences least often (2.4 for LVF, 2.6 for RVF), and low cloze sentences slightly more often (2.5 for LVF, 2.7 for RVF). There was a numerical trend for RVF items to be recognized more than LVF items but this difference did not reach significance [*F*(1,21) = 2.06], nor did VF interact with cloze bins [*F*(2,42) = 0.13].

### ERPs

**Figure 1:**
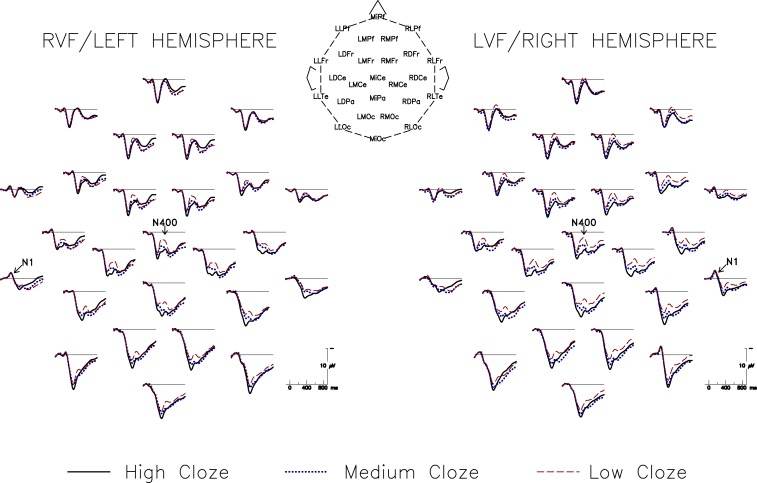
**Grand average ERPs for both visual fields at all electrode sites, for High, Low, and Medium cloze probability sentence completions**. Both hemispheres are sensitive to contextual information in sentence contexts and, as expected, N400 amplitudes for both VF/hemispheres monotonically decrease with increasing constraints.

Sentence-final ERP averages for High, Medium, and Low Cloze conditions are plotted in Figure 1 for each of the 26 scalp sites as arranged on the head, for both VFs. Between 0 and 250 ms post-stimulus onset, all conditions evoke a series of brain potentials typical of visual stimulation. Over lateral posterior sites, sensory components (e.g., the N1) are lateralized and contralaterally delayed; after about 300 ms, all conditions produce a contralateral posterior selection negativity, indicative of initial unilateral reception and sustained processing bias induced by hemifield stimulation. Beginning around 250 ms, a broadly distributed negative-going wave, the N400, varies in magnitude by condition. As expected, both VF presentation conditions elicit monotonically decreasing N400s as cloze probability increases.

#### Overall N400 effects

**Figure 2:**
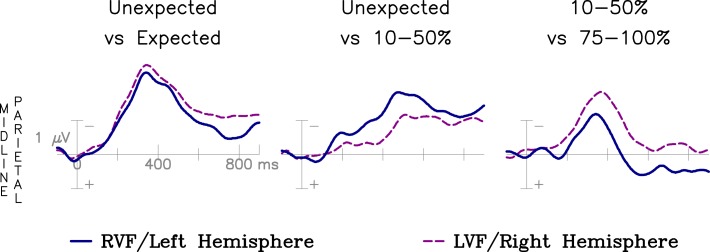
**Difference wave comparisons across VF of presentation**. The overall N400 expectancy effect (Unexpected versus 90–100% cloze) is similar in size and timing across VF of presentation. The difference between Unexpected endings and Weakly Expected endings (10–50% cloze) is larger for the RVF/LH, and the difference between Weakly Expected endings and Strongly Expected endings (75–100% cloze) is larger for the LVF/RH.

The effect of contextual constraint on N400 amplitude as a function of VF of stimulation is the focus of this report. To demonstrate that basic results replicate past work, the overall N400 effect (for Strongly Expected items, 90–100% cloze versus Unexpected items, 3% cloze) was examined with a point-by-point subtraction across the epoch for each VF. The resulting difference waves (displayed in Figure 2) were used to measure peak latency of the effect at the six central-parietal electrode sites known to produce maximal N400 effects (peak negative amplitude in a 200–500 ms window, measured with a bandpass filter of 0.2–10 Hz). A repeated-measures ANOVA [2 (VF) × 6 (Electrode)] revealed no significant difference in the latency of the N400 effect [*F*(1,23) = 0.05; LVF = 342.4 ms, SEM = 7.04 ms; RVF = 337.9 ms, SEM = 6.56 ms] and no interaction with Electrode site [*F*(5,115) = 1.10].

The magnitude of the effect was measured as the mean amplitude in a 250–450 ms time window (approximately centered around the peak latency of the effect) over all electrode sites^1^. Again, no significant effect of VF was observed [*F*(1,23) = 0.49; LVF = −3.19, SEM = 0.13; RVF = −2.71, SEM = 0.13], nor did the effect of VF on the magnitude of the N400 difference depend on Electrode site [*F*(25,775) = 0.58]. These results are consistent with all past studies of the effect of hemifield stimulation on the overall N400 patterns (see, e.g., Federmeier and Kutas, 1999a; Coulson and Williams, 2005; Coulson et al., 2005; Federmeier et al., 2005; Wlotko and Federmeier, 2007; reviewed in Federmeier et al., 2008), suggesting that differences in patterns of brain activity across VF are not due to a simple latency shift or disparities in overall magnitude of response.

#### N400 responses for weak contexts

In Wlotko and Federmeier (2007), responses across VFs were most different for weakly expected sentence completions. First, to directly replicate that study with traditional ERP analyses, we created ERP waveform averages that were most similar to the conditions in that study. Strongly Expected Endings consisted of a cloze range of approximately 75–100% and Weakly Expected endings consisted of a cloze range of approximately 10–50%. The Unexpected endings were similar to the Weakly Constrained Unexpected condition in Wlotko and Federmeier (2007). The 50–75% cloze range was not represented in Wlotko and Federmeier (2007) and the Strongly Constrained Unexpected condition was not included in the present study. N400 mean amplitudes for each condition were measured in the 250–450 ms time window over all electrode sites. To directly compare N400 effects across VF of presentation, difference waves were formed for effects of interest and the amplitudes of the resulting waveforms were measured in the same time window and over the same electrode sites.

Wlotko and Federmeier (2007) found that the effect of expectancy for Weakly Constraining contexts was larger for RVF than LVF presentation. To replicate this effect, N400s for sentence endings of 10–50% cloze were compared to N400s for Unexpected endings. This difference was significant for RVF [*F*(1,23) = 13.93, *p* = 0.001] but not LVF [*F*(1,23) = 2.55, *p* = 0.12; interaction with Electrode, *F*(25,575) = 0.58] presentation. When the size of this effect is compared across VF, the N400 effect is larger for the RVF than the LVF, as it was in Wlotko and Federmeier (2007) [*F*(1,23) = 27.67, *p* < 0.001].

Wlotko and Federmeier (2007) also found that the effect of Constraint was larger for LVF than RVF presentation. In the current study, the difference between the 10–50% cloze bin and 75–100% cloze bin was significant for both VFs [RVF, *F*(1,23) = 10.81, *p* = 0.003; LVF, *F*(1,23) = 31.95, *p* < 0.0001], and this effect was significantly larger for the LVF, as it was in Wlotko and Federmeier (2007) [*F*(1,23) = 4.78, *p* = 0.039]. These effects are displayed in Figure 2.

These analyses replicate the findings from Wlotko and Federmeier (2007) suggesting that LVF/RH weakly expected items receive little N400 facilitation compared to unexpected endings, whereas the RVF/LH shows a greater degree of N400 facilitation for weakly expected items.

#### Modeling N400 responses as a function of cloze probability

To explicitly take advantage of the continuous manipulation of sentential context employed in the design of this study, cloze probability was regressed on the measured N400 amplitude over each of the 300 items for each participant. To take into account the repeated-measures aspect of this analysis, a hierarchical linear model (or multilevel) model was used, considering each participant’s amplitude measurements to be “nested” within that participant. This procedure allows statistically efficient estimation of the regression parameters, provides correct standard errors for fixed effects, and allows an examination of random effects (across participants, in this case). Thus, in contrast to grand average correlations or single-item analyses that do not take between-subject variability into account, the HLM procedures provide inferential statistics at the appropriate level (see Wlotko and Federmeier, 2012a for a comparison of HLM and single-item analyses of ERPs).

The N400 was parameterized as the mean amplitude in the 250–450 ms window, over the six central-parietal channels where N400s are known to be largest. Thus, a case in the hierarchical analysis consisted of a single-trial amplitude and associated cloze probability for that item, along with its corresponding VF of presentation for that participant. Cloze probability was coded as a proportion from 0 to 1, such that the intercept in the model would represent the predicted amplitude for a 0% cloze item (a wholly unexpected word) and the regression parameter would correspond to the expected effect of a 100% cloze item (completely expected) compared to the intercept value.

The model fitting procedure followed Wlotko and Federmeier (2012a) using PROC MIXED in SAS 9.2. A model estimating the effect of cloze probability on N400 was fit to the data, including a random intercept for participants to allow for the hierarchical structure of the data. In all cases, examination of information criteria (in particular, the Bayesian Information Criterion, BIC; Schwarz, 1978) suggested the use of random effects best fit the data. Thus, all parameters presented below are from models incorporating random effects across participants (exploring other random structures revealed little influence on the fixed effect parameters).

The overall effect of sentence-level expectancy was estimated as 3.98 [95% confidence interval, based on a t distribution with 22.7 degrees of freedom [3.07, 4.90]; *p* < 0.001]. Corroborating the ANOVA reported above, models including VF or an interaction between cloze probability and VF did not reveal a difference in the overall effect of cloze probability as a function of VF. Thus, both hemispheres elicit decreasing N400s in response to increasing constraint, within a similar range of N400 amplitude (about 4 μV across the entire range of constraint).

A central goal of the current study was to test the hypothesis that despite the overall similarity in the size and timing of the N400 across VF, both hemispheres depart from the typical evenly graded N400 response as a function of cloze probability. To test this hypothesis, a quadratic term for cloze probability was added to the model, as well as the interaction between VF and the quadratic term. The quadratic term itself was marginally reliably different from zero (coefficient = −2.10, *p* = 0.06), and the interaction term was significant (coefficient for interaction of VF and quadratic term = 4.30, 95% CI [1.65, 6.95]; *p* = 0.002). VF was coded as a categorical variable, so that the quadratic term alone reflects the quadratic component for the LVF, and the interaction represents the additional quadratic component for the RVF. The inclusion of the quadratic terms caused the interaction of VF and the linear term to also become significantly different from zero. Examination of BIC suggested the quadratic model was a better fit to the data than the linear-only model. However, adding a cubic term to the model did not explain significantly more variance, nor did a cubic term interacting with VF.

**Figure 3:**
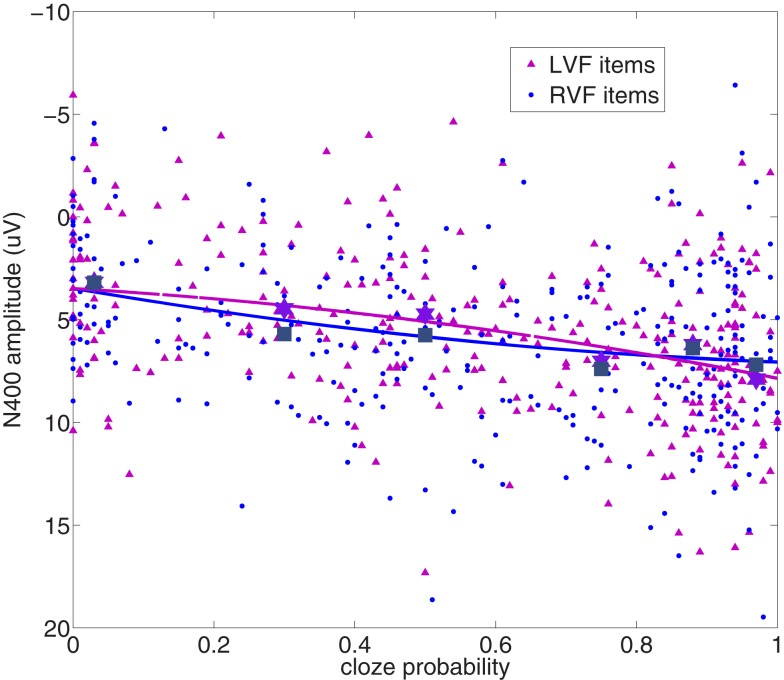
**Item-level N400 amplitudes (averaged across participants) plotted against cloze probability for all items in each VF**. Grand mean amplitudes for the 6 cloze bins described in the Method section are overplotted (dark blue squares: RVF/LH; purple stars: LVF/RH) along with the predicted response functions from the hierarchical model.

**Figure 4:**
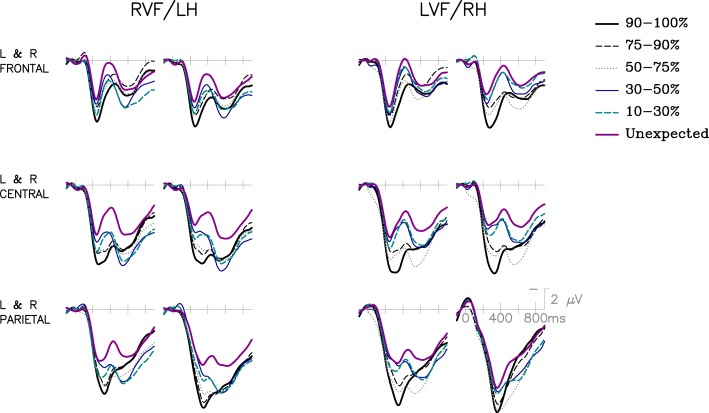
**Traditional grand average ERPs for the 6 cloze probability bins described in the Method section at Left and Right Frontal, Central, and Parietal sites (LDFr, RDFr, LMCe, RMCe, LDPa, RDPa)**.

Table 2 presents the parameters from both the linear and quadratic HLM models. The quadratic model parameter estimates lead to predicted equations of LVF = 3.5 + 2.14*(cloze) + 2.2*(cloze^2^) and RVF = 3.5 + 5.67*(cloze) − 2.1*(cloze^2^). The opposite (and significantly different) signs for the quadratic components for LVF and RVF accounts for the difference in the responses of the two hemispheres as a function of cloze, particularly over weak to moderately constrained items (as also demonstrated by the pairwise comparisons in the previous section). These response functions are shown in Figure 3, which presents item-level ERP amplitudes (collapsed over participants) plotted as a function of cloze probability. The predicted functions for the two VFs and the grand means for the six 50-item bins described in the Method section are overplotted with the item-level data^2^. RVF items are consistently more positive (smaller N400) than LVF items, particularly in the middle of the cloze probability range, a difference modeled by the negative quadratic component in the RVF regression equation. The traditional ERP grand averages for the six cloze bins are displayed in Figure 4.

**Table 2:** **Relevant HLM parameters and fit statistics for N400 data predicted by cloze probability as given by SAS PROC MIXED**.

	Intercept (SE)	Variance of intercept (SE)	Cloze effect (SE)	Variance of cloze effect (SE)	VF interaction (SE)	Variance of residuals/error term (SE)	Deviance [−2*log(like)]	AIC	BIC
Null model (no predictors)	5.78 (0.62)	9.23 (2.73)	–	–	–	106.1 (0.83)	246943	246947	246949
Linear cloze effect	3.55 (0.57)	7.40 (2.27)	3.98 (0.44)	3.99 (1.39)	−0.13 (0.11)	103.9 (0.81)	246289	246297	246301
Quadratic cloze effect	3.51 (0.63)	10.32 (3.36)	−2.10 (1.12)	1.23 (0.55)	4.30 (1.33)	103.1 (0.81)	246177	246183	246186

*Data were coded so that each parameter represents the LVF effect, and the interaction represents the additional effect for RVF. HLM fixed effects (i.e., intercept, linear cloze, quadratic cloze, VF interaction) are presented, and the estimated variance of the effect (i.e., the random portion) is presented along with standard errors in parentheses. Fit statistics include deviance (−2 times the natural log of the likelihood value), Akaike Information Criterion (AIC), and Bayesian Information Criterion (BIC)*.

A model with a quadratic term but no interaction with VF is equivalent to a model averaging over the two VFs; this model was fit to the data to determine whether any deviation from a linear response could be detected. In this “averaged LVF and RVF” model, the quadratic term is not significant. A detailed analysis of N400 responses to the same (centrally presented) sentence materials was conducted in Wlotko and Federmeier (2012a). When data from that study were fit to the quadratic model, a highly significant linear component was found, as expected and reported in Wlotko and Federmeier (2012a), but the quadratic term was not significant.

#### Later (600–900 ms) effects

Visual inspection revealed that there may be condition-related differences in the post-N400 time window for both RVF/LH and LVF/RH presentation. As we had no *a priori* hypotheses regarding how later effects across the two hemispheres would covary with a continuous manipulation of cloze probability, the data in this time window were not modeled using a regression approach as for the N400. Instead, mean amplitudes from the 600–900 ms time window were submitted to a 2 (VF) × 6 (Cloze Bins) × 26 (Electrode Site) repeated-measures ANOVA. A main effect of Cloze was observed [*F*(5,115) = 2.96, *p* = 0.015, ε = 0.942] as well as an interaction between Cloze and Electrode [*F*(125,2875) = 3.90, *p* < 0.001, ε = 0.127]. The main effect of VF was not significant [*F*(1,23) = 1.31], nor were the interactions involving VF and Cloze (*F*s < 1.64), although VF did interact with Electrode Site as expected based on the posterior contralateral selection negativity typical of unilateral presentation of stimuli [*F*(25,575) = 47.83, *p* < 0.001, ε = 0.205].

The patterns of ERP waveforms suggest that there may be different condition-related effects at different scalp sites, potentially supported by the Cloze × Electrode interaction. As the moderately to strongly constraining items appeared more negative than the other conditions for both RVF/LH and LVF/RH presentation, we tested the same contrast over the same time window and electrode sites as in Wlotko and Federmeier (2012b) to determine whether a similar pattern was observed with lateralized presentation. Mean amplitudes for the moderately strong constraint condition (75–90% cloze) and the unexpected condition were computed over 600–900 ms at LDFr, LMFr, and LDCe. There was no main effect of condition [*F*(1,23) = 0.65] nor an interaction with VF [*F*(2,46) = 0.22]. Thus, the effect described in Wlotko and Federmeier (2012b) appears to be diminished with lateralized presentation.

Finally, posterior effects were analyzed with the medial central-posterior channels (LMCe, MiCe, RMCe, LDPa, MiPa, RDPa, LMOc, MiOc, RMOc) in the same 600–900 ms time window. A 2 (VF) × 6 (Cloze Bin) × 9 (Electrode Site) repeated-measures ANOVA revealed a main effect of Cloze [*F*(5,115) = 4.02, *p* = 0.002, ε = 0.937], a marginal main effect of VF [*F*(1,23) = 3.45, *p* = 0.076, but no interactions between Cloze and VF (*F*s < 1.09). As this effect likely represents the commonly observed but currently not well specified post-N400 late positive complex (LPC), pairwise comparisons were performed on the extreme cloze conditions of 90–100% vs. Unexpected (cf. Wlotko and Federmeier, 2007). This effect was significant for LVF/RH presentation [*F*(1,23) = 4.96, *p* = 0.036] and marginal for RVF/LH presentation [*F*(1,23) = 3.14, *p* = 0.089], but the size of the difference was not significantly different across the VFs/hemispheres [*F*(1,23) = 0.44]. Thus, asymmetric late effects were not observed in this study.

## Discussion

A necessary beginning to elucidating the neural mechanisms of language is understanding the capabilities and specializations of each hemisphere for subcomponents of comprehension by, for example, studying the abilities of patients with unilateral damage. Thus, research on laterality has often focused on processes or domains for which one hemisphere is “dominant” or “specialized.” In language, LH advantages (and deficits for patients with LH lesions) in tasks requiring phonological or syntactic processing have been taken to indicate LH specialization for these basic language processes. The RH shows advantages (and deficits in patients) for other types of tasks, for example, those relying on discourse or prosodic processing. However, normal comprehension typically proceeds with both hemispheres working in concert. Thus, a more complete description of neurocognitive mechanisms supporting language requires an appreciation of the *joint* contributions of the two hemispheres to processes important for comprehension.

Our prior work has shown that when processing is biased to one hemisphere, via half-field stimulation, LVF/RH, and RVF/LH patterns of ERP responses to variations in sentential context are different from each other – and, critically, different in each from patterns obtained with central presentation. These studies have focused on the N400 component of the human event-related brain potential, a direct, sensitive, and powerful tool for investigating the use of semantic memory during normal language comprehension. Our results suggest that typical comprehension processes may arise out of a combination of distinct processing biases in the two hemispheres, neither of which on its own generates the typically observed response (Wlotko and Federmeier, 2007; reviewed in Federmeier et al., 2008).

Here we extend the prior work by using a continuous variation of sentential context – i.e., sentence constraint, defined by cloze probability – rather than a categorical one. This design offers a more fine grained examination of the response to contextual information by the hemispheres than has previously been considered. The results directly replicated the categorical manipulation of sentence constraint and expectancy in prior work (Wlotko and Federmeier, 2007). Furthermore, the item-level analysis of ERP data across the full range of constraint – offering a fuller picture of hemispheric sensitivity to sentential context than has been presented to date – produced a statistically significant deviation from the evenly graded (linear) relationship found for central presentation, in the form of a quadratic term that differed as a function of VF of presentation. Taken together, these results support the idea that weak sentential contexts in particular are differently processed by the hemispheres.

These data provoke the idea that the N400 reflects unique contributions from the two hemispheres during normal comprehension, which can lead to a new way of thinking about the underlying neural processes that give rise to the scalp-recorded potential. Although multiple distributed brain areas have been implicated in the generation of the N400, it is nevertheless typically characterized as a unified process that unfolds in response to semantically meaningful stimuli (Van Petten and Luka, 2006; Federmeier and Laszlo, 2009). Such a unified process may be expected to arise within a single hemisphere, meaning that patterns seen with central presentation would resemble that of one of the hemispheres as probed through lateralized presentation; or, alternatively, through (perhaps redundant) activity arising across both hemispheres, leading to the prediction of similar responses across central presentation and lateral presentation to either hemisphere. Even frameworks that place more emphasis on hemispheric communication and cooperation have tended to assume either that the mode of one hemisphere dominates processing, using some resources from the other hemisphere when hemispheric cooperation is induced; or that the hemispheres unify computations to (singly) perform a particular task (see, e.g., Hellige, 1995; Banich, 1998; reviewed in Liederman, 1998).

The data presented here, instead, suggest that the N400 seen for centrally apprehended words during normal language comprehension may reflect an “average” of the processing modes of each hemisphere. Thus, each hemisphere may individually respond to available contextual information with its own processing biases and contribute those responses as comprehension proceeds. That is, rather than a unified response, the hemispheres elicit distinct patterns of N400 activity that, under non-hemispherically biased conditions, appear as a summation of the two.

Another possibility is that the scalp activity *per se* does not reflect a mere average of LH and RH activity, but that the two hemispheres coordinate activity when hemispheric cooperation is more likely, as in central compared to lateral presentation. In fact, it is likely that the N400 observed with central presentation reflects a larger contribution from the LH than the RH (Van Petten and Luka, 2006). So instead of a “LH” N400 and a “RH” N400 that results in an unweighted averaged potential at the scalp, perhaps the processing biases of the hemispheres shape the activity of the entire (bihemispheric) N400 network to result in the graded response, and it is mainly when processing is shifted to one hemisphere or the other (e.g., during lateral presentation) that the processing modes of the two hemispheres can be observed individually.

Increasing facilitation of word processing with increasing contextual support is one of the most robust findings in cognitive science. Indeed, a substantial literature across methodologies shows a graded response to cloze probability (see, e.g., Simpson, 1991). However, there are several examples in the literature demonstrating that examining one summary aspect of a dataset can obscure the true underlying distributions (e.g., Van Zandt and Ratcliff, 1995). Failing to consider that the two hemispheres may respond differently in a particular set of circumstances may, in this case, lead to the characterization of a response that no part of the brain itself generates but that emerges as a combination of outputs from the two hemispheres. It is important to point out, however, that even though the N400 pattern recorded at the scalp may reflect individual processing biases of the two hemispheres, at some point in processing this information seems to be combined in service of cognitive operations. Lexical decision RTs seem to track cloze probability in a graded way, similar to the classic N400 pattern (Schwanenflugel and LaCount, 1988), as do eye tracking measures (e.g., Ehrlich and Rayner, 1981). These patterns may arise if, for example, the processes needed for lexical decision “read out” the average of the activity from the two hemispheres as part of the decision process.

Later stage comprehension processes have only recently come under systematic scrutiny in the ERP literature. Several different types of post-N400 effects have been described. For example, a late frontal positivity is regarded as an index of disconfirmed predictions in language comprehension, as it is enhanced for unexpected (but plausible) completions of contexts that are strongly constraining. Intriguingly, this effect was not observed with lateralized presentation of the critical stimuli in Wlotko and Federmeier (2007). Similarly, for central presentation of the stimuli in this study, a left-frontal negativity was associated with a reinterpretation of contextual information when multiple interpretations of the context were likely (Wlotko and Federmeier, 2012b). As for the frontal positivity effect, the left-frontal negativity appears to be diminished with lateralized presentation, as it was not robustly elicited in the current study (though both RVF/LH and LVF/RH ERPs generally fit the pattern observed in Wlotko and Federmeier (2012b), with the moderately constraining items eliciting the most negative-going ERPs at left-frontal channels).

Late comprehension processes may be resource-demanding, perhaps requiring working memory and/or sustained attention to the contextual material as context unfolds. Lateralized presentation may disrupt such processes by hindering hemispheric cooperation needed for the mechanisms to be engaged. Another possibility is that more resources are devoted to perceiving words in the periphery, affecting allocation of resources to later stages. Clearly, further work is needed to clarify the functional specificity of the several types of post-N400 effects in general, as well as the hemispheric contributions to these processes.

Regardless of the exact configuration of neural resources engaged during the elicitation of these ERP components, the differing hemispheric patterns of N400 response functions to sentence constraint help to provide more insight into how the two hemispheres may employ distinct modes of comprehension in parallel during normal message-level comprehension. Given the close association between the N400 and initial access to semantic memory (Kutas and Federmeier, 2011), the unique response functions for the two hemispheres imply that semantics is not singly “looked up” or computed within one brain area or even within one hemisphere, as has been assumed in some models of word recognition (e.g., Forster, 1999; Cohen et al., 2000; Coltheart et al., 2001).

Simultaneous implementation of different processors may permit the hemispheres to trade off benefits and consequences of their respective modes. For example, although we consistently find that both hemispheres are able to process message-level information, the RH requires a larger base of contextual support (greater than 50% cloze probability) in order to evidence as much facilitation from the context as the LH. We have proposed that the LH “over-facilitation” for weak contexts arises out of the LH tendency, putatively based on its more efficient top-down connections between frontal and temporal brain regions, to use contextual information to predict – that is, to pre-activate likely upcoming stimuli based on the context (Federmeier, 2007; Federmeier et al., 2008). As cloze probability is a measure of the constraint – or predictability – that a sentence context can provide, our results appear to show that the LH can take advantage of predictability, even when constraint is somewhat weak (10–50% cloze probability).

Interestingly, corresponding results appear at the word level, using lexical associates of moderate strength. Association norms are similar to cloze probabilities, as the strength of association is calculated by asking participants to produce the first word that comes to mind when hearing or reading the prompting stimulus and then tallying the distribution of responses (analogous to the cloze probability calculation). When presented in the RVF/LH, moderately associated targets are primed more than those same targets presented in the LVF/RH, as measured by N400 amplitude reduction (Kandhadai and Federmeier, 2010). Thus, the LH advantage when even a moderate amount of predictability is available appears at both the word and sentence levels. In addition, there is further evidence that this phenomenon is not limited to the visual modality, as similar results were observed in a dichotic listening task: behavioral responses to critical words were facilitated to a similar degree when strong and weak contexts were presented to the right ear/LH channel, whereas only strong contexts facilitated responses for the left ear/RH channel (Aydelott et al., 2012).

The two modes of comprehension employed by the hemispheres may be advantageous across different processing circumstances. A predictive strategy, employed by the LH, can provide benefits in the form of pre-activation for features of possible upcoming stimuli that will ease processing when those predictable stimuli are then encountered. These benefits seem to accrue even when the context is only moderately predictive. In contrast, the reliance on stronger context to provide facilitation for the RH may make reinterpretation easier for less predictable concepts or when the context is weak, as the LH predictive strategy may (through additional mechanisms downstream from the N400) suppress, inactivate, or otherwise fail to maintain non-predictable words/concepts within sentence or discourse processing, especially when strong predictions are made. That is, a predictive strategy may engender selecting or settling into one particular representation, especially when predictions are confirmed. In this case, all of the potential advantages of predictive processing are realized. On the other hand, when new information requires a revision or reinterpretation of the representation, this may be easier when strong predictions were not generated in the first place, as for the RH mode of processing. This asymmetry in use of context may lead to the well-documented specialized role of the RH in discourse processing (cf. Federmeier and Benjamin, 2005), in some types of non-literal language (e.g., processing of jokes, cf. Coulson and Williams, 2005), and in other cases in which words or concepts may become important downstream from their initial occurrence.

The perspective that each hemisphere differently makes use of contextual information may have implications for recovery from aphasia or for language deficits acquired after RH injury, as damage to each hemisphere can disrupt the use of context information in unique ways (cf. Grindrod and Baum, 2003). Further, this type of framework may also prove illuminating for examining language processing in disorders associated with abnormal laterality such as autism or schizophrenia. Healthy aging, too, has been shown to render prediction during online comprehension less likely, and indeed, less N400 facilitation is observed in older adults for weak contextual information (Wlotko and Federmeier, 2012a; Wlotko et al., 2012), similar to the RH pattern observed here. In all of these cases, the balance of hemispheric processing may be shifted relative to the healthy young adult brain. Thus, considering the distinct contributions of the hemispheres is required to fully elucidate cognitive and neural mechanisms underlying the differences seen in these populations.

Furthermore, the asymmetric pattern of N400 responses to sentential context places constraints on how predictive language mechanisms may manifest in the brain. For instance, the finding that the LH can take advantage of even weakly predictive context information argues against the notion that predictions could only be useful for the brain in unusually highly constraining contexts, making a predictive strategy unlikely to benefit processing in the course of normal language use (e.g., Jackendoff, 2002). Investigations of the consequences of predictive processing during language comprehension have revealed that constraining contextual information can lead to the pre-activation of semantic, grammatical, phonological, and orthographic information about specific lexical items (Federmeier and Kutas, 1999b; Wicha et al., 2004; DeLong et al., 2005; Van Berkum et al., 2005; Laszlo and Federmeier, 2009). The well-studied graded facilitation across variation in constraint is compatible with several ways of characterizing the anticipatory use of context during online language processing. Constraint could affect the strength of probabilistic predictions, the number or amount of pre-activated features matching or mismatching those of the best completion, the chance of building a successful specific prediction, or even the likelihood of generating a prediction at all (see, e.g., Schwanenflugel and LaCount, 1988; DeLong et al., 2005; Thornhill and Van Petten, 2012; Van Petten and Luka, 2012). The LH N400 pattern in this study implies that the intermediate response to weakly expected endings (during central presentation) does *not* arise purely out of the mixture of some correctly predicted and some incorrectly predicted endings in the weak condition across trials in the experiment. That is, the intermediate facilitation for weakly constraining items could be observed at the average level if participants’ brains initiated a strategy to form strong, specific predictions on every trial. In that case, some of the time participants would correctly predict the best completion presented in the experiment and in other instances they would predict a different completion from the one that had been presented. If that were the case, however, the LH response should resemble the canonical graded pattern and the over-facilitation for weak contexts for RVF/LH presentation should not be observed.

While the LH presumably cannot successfully predict the upcoming sentence-final word in all or even most weakly constraining sentences, an *attempt* at prediction in these cases will bring on the same types of top-down mechanisms involved in the pre-activation of features of predictable words in strong contexts. In our framework, these mechanisms are production-like processes (see Federmeier, 2007) that are likely to activate the same types of information/features as used by the participants in the cloze task to generate a response in a particular context. Thus, the best completion we present in the experiment is likely to match at least some of the (semantic) features brought online during predictive comprehension. The featural match at the semantic level would result in the N400 facilitation observed for weak contexts. Thus, as long as predictions are not disconfirmed, any level of predictability can be beneficial to the LH. Unless a response is forced (i.e., in the cloze task), the weakness of the context may not lead the LH to successfully pre-activate one lexical item, and thus, no consequences of prediction are observed in the weakly constraining contexts when unexpected items are presented (i.e., the late frontal positivity downstream from the N400). By contrast, the RH, lacking strong top-down production-like mechanisms, should not pre-activate features of possible upcoming words as the LH does. As such, integrating a weakly expected item into the current message-level representation, which by definition has little semantic support for the sentence completion, is more similar to integrating an unexpected item and less N400 facilitation is observed.

Thus, the N400 is not a unitary response that simply responds probabilistically to contextual information. Instead, the processing modes of the two hemispheres jointly shape the way context is used during comprehension. That is, rather than a simple feed-forward, staged processing stream wherein visual information across the two hemispheres is integrated and subjected to the processing specialties of a particular hemisphere, the two hemispheres contribute to semantic comprehension processes simultaneously. This type of architecture can result in multiple patterns of semantic activation rather than just one outcome, at least by the time of the N400. The LH bias toward predictive processing leads to facilitation for words embedded in weak contexts, whereas the RH reliance on context to integrate word meanings is less efficient at facilitation when context is weak. That the comprehension system is divided across the two hemispheres may mean that there is not “the” N400, but that neural activity typically observed in the system reflects multiple processing modes implemented in parallel. These are important and potentially wide-reaching considerations when attempting to build a model of how language comprehension unfolds in a large network of brain areas, distributed within and across the two cerebral hemispheres.

## Conflict of Interest Statement

The authors declare that the research was conducted in the absence of any commercial or financial relationships that could be construed as a potential conflict of interest.

## Footnotes

^1^Visual inspection of the waveforms reveals that there may be condition-related differences in an earlier time window, possibly on the P2 component. However, the pattern of increasing positive amplitudes with increasing cloze probability in this time window is not distinct from the pattern in the N400 time window. N400 effects in visually presented sentence comprehension paradigms can onset as early as 200 ms post-stimulus onset (Van Petten and Luka, 2012). Furthermore, there is no evidence that any effects of cloze have a distinct scalp distribution in the earlier time window. Most importantly for the present study, there is no interaction between cloze range and VF when measured in the 180–280 ms time window in the five medial frontal channels where P2 effects have been previously observed [F(5,115) = 0.79], although the main effect of Cloze is significant [*F*(5,115) = 7.95, *p* < 0.001, ε = 0.899]. Thus, whether these differences reflect the early part of the N400 or P2-related activity, there is no modulation of overall ERP patterns by VF of presentation, which is the focus of this report.

^2^When the regression equations are fit separately to LVF and RVF data, a similar amount of variance is explained in each: 13.3% of the variance for RVF and 13.6% for LVF (cf. 17% for central presentation N400 data in Wlotko and Federmeier, 2012a).
